# Fellow-Workers and Their Doings

**Published:** 1914-07-18

**Authors:** 


### ROUND THE WORLD.

### Fellow-Workers and their Doings.

[The Editor Invites Early Information and Contributions Marked "Fellow-Workers."]

Sir Cooper Perry, who has recently given up general medical work at Guy's, is by no means severing his connection with that institution, for he is continuing as superintendent thereof, as well as maintaining his appointment of physician to the skin department. It is owing to the fact that the former office takes up so much of his time that he has decided to relinquish the post of physician, which he has held for some years. Sir Cooper Perry has done a great deal for the hospital world as a member of the General Council and Distribution Committee of King Edward's Hospital Fund, and for his public services received the honour of knighthood in 1903; in early years he was a student of the London Hospital, subsequently attaching himself to Guy's, where he has had a very distinguished career.

A useful aid to *x*-ray examination of the abdomen has been devised by Mr. E. W. H. Shenton; this consists of a wide lead glass cylinder attached to a fluorescent screen. The object of the instrument is to obtain a closer view of intra-abdominal organs, a result which is secured by pressing the glass tube well into the abdominal wall, so that an intensified view is obtained of the small area within its circumference. In describing this invention, which he calls "An X-ray Screen Compressor," to the *Lancet* (July 4, 1914), Mr. Shenton claims that it is particularly useful in the detection of renal calculus. The work of this well-known radiologist, who is senior radiographer at Guy's Hospital, has done much to advance the usefulness of *x*-ray methods.

Dr. Arthur Stanley Barnes, who has recently gained promotion to the senior staff at the General Hospital, Birmingham, where he has for some time been assistant physician, obtained the main part of his medical education in Birmingham, taking the degrees of M.B., B.Ch., and D.Sc. of the local university. He is also an M.D. Lond., having taken double honours at the M.B. examination, and an M.R.C.P. Lond. In addition to these successes, Dr. Barnes won the Sands Cox Scholarship and an entrance science scholarship in 1892; while three years later he was awarded first-class honours in physiology at the B.Sc. London. His original work has evinced special interest in nervous disorders, having been largely concerned with investigations in connection with neuritis and various spinal degenerations.

Mr. A. Hopewell-Smith, who is to be congratulated on his appointment as Professor of Comparative Odontology and Dental Histology in the University of Pennsylvania, is a member of the staff at the Royal Dental Hospital in Leicester Square, and has distinguished himself by taking the John Tomes Prize of the Royal College of Surgeons. Qualifying as a medical practitioner, M.R.C.S., L.R.C.P., as well as a dental licentiate of the Royal College of Surgeons, Mr. Hopewell-Smith, who did his clinical work at Charing Cross Hospital, has for some years been a keen investigator of dental pathology, and has contributed numerous books and articles on his subject. In addition to his London appointments, he is examiner in dental surgery to the Universities of Leeds and Liverpool. The chair to which he has been appointed is a new one, and it is a tribute to British dental surgery that its occupant should have been chosen from London—a point which may be noted with advantage by the British public, which in these days is somewhat inclined to give pre-eminence to American methods of dentistry.

As is well known, the use of cocaine for anæsthetising the larynx is not always entirely satisfactory; consequently, Dr. Courtenay Yorke's development of a better method of local laryngeal anæsthesia is very important. Communicating his results to the *British Medical Journal* (June 13, 1914), this laryngologist advises the injection of novocain around the nerves which supply the larynx, in addition to painting the throat with a solution of cocaine; at the same time a preliminary injection of morphine and atropine is advocated. Dr. Courtenay Yorke is an M.D. Liverpool, M.B. Lond., and F.R.C.S. Eng.; his work in this particular field has been carried out at the Liverpool Consumption Hospital, to which he is now honorary laryngologist.

Dr. D. Henriques De Souza, the new demonstrator in physiology at King's College, is a member of the staff of Westminster Hospital, where he is now senior assistant physician and joint lecturer in medicine. Formerly a student of University College Hospital, Dr. De Souza speedily became recognised as a scientific investigator of physiological problems, and obtained the important post of demonstrator of physiology at Sheffield University. In addition to being an M.D., M.R.C.P. Lond., he holds the degree of Doctor of Science, and was awarded first-class honours in physiology at the London B.Sc. examination.

Mr. Sydney Stephenson has lately had a good deal of work as Secretary of the recent Ophthalmological Congress at Oxford. Well known as an indefatigable worker in ophthalmology, Mr. Stephenson is one of a comparatively few specialists who hold the recently constituted diploma in ophthalmology of Oxford University. His post as ophthalmological surgeon to the Queen's Hospital for Children has naturally led him to devote particular attention to the eye troubles of early years, although he has made equally important observations on the ophthalmic disorders of adult life. It will be remembered that a few years ago Mr. Stephenson was awarded the Middlemore prize in ophthalmology by the British Medical Association.

Considering how much energy must be wasted by ill-fitting boots and shoes, it is remarkable that our proper requirements in footgear have been so much ignored by the medical profession. Certainly Mr. E. Muirhead Little's paper on "Boots and Shoes from Historical and Surgical Points of View" (the *Lancet*, June 20, 1914) raises many points of importance which may be properly studied by surgeons and medical practitioners who have the care of growing children. Mr. Muirhead Little, who is senior surgeon to the Royal National Orthopædic Hospital, traces the development of footgear from very early times, and deals with shoes which, he tells us, were made some 3,000 or 4,000 years ago, as well as with those of intermediate ages down to the present time.

